# The Health of India

**Published:** 1904-12-31

**Authors:** 


### THE HEALTH OF INDIA.

A medical report has just been issued on the health of India during 1902. It deals not only with the health of the European and Native troops, of the prisoners in gaols, and of the general population, but also with vaccination, medical institutions, including medical schools, lunatic asylums, and sanitary works.

### European Army.

The health of the European troops serving in India during the year 1902 was, on the whole, satisfactory; the admission, daily sick, and invaliding rates were slightly lower, but the death-rate was higher by 1 per 1,000. Bombay was the most unhealthy of the four Commands, Bengal coming next, and both were more unhealthy than in the previous year. The chief causes of sickness in the whole European Army were, as usual, ague and venereal diseases, the latter yielding a ratio of 281 per 1,000, against 276 in 1901, and the former 247 per 1,000, against 293. Forty-nine per cent. of the total sickness resulted from these two causes. Enteric fever, as usual, was the chief cause of mortality, the death-rate from it being 4·29, or nearly 1 per 1,000 higher than the ratio of the previous year. Of the total deaths from enteric fever nearly 87 per cent. occurred in men between the ages 20-30. There was again very little cholera among the European troops, only 3 cases having been recorded during the year against 12 cases in 1901. In both years all the cases were fatal.

Venereal diseases ranked first in the list of causes of admission to hospital and first in the cause of invaliding. They caused one-quarter of the total sickness during 1902. The admission rate per 1,000 strength from venereal diseases was 5·4 higher than in 1901, thus interrupting the steady yearly decrease which commenced with the year 1896. The slight increase is attributed chiefly to the arrival of a number of regiments or regimental drafts from field service in South Africa. The number of men constantly sick in hospital from these diseases was 1,431. As regards the influence of age and length of residence in India upon invaliding 35 per cent. of the total number invalided were under 25 years of age, and 48 per cent. were of less than five years' service.

### Native Army.

The health of the Native Army of India was on the whole better than in 1901, though the death-rate was slightly higher. The chief causes of sickness in the Native Army was ague, which contributed 38 per cent. of the total admissions. Among the diseases from which the admissions to hospital were higher than in the previous year were small-pox, enteric fever, tubercle of the lungs, and pneumonia; while from influenza, cholera, respiratory diseases other than pneumonia, diarrhœa, and venereal disease, there was a decrease in the admissions. The admissions from venereal diseases were 33 per 1,000 of strength, as compared with 281 per 1,000 among European troops. Pneumonia was, as usual, the chief cause of the deaths in the Native Army, and contributed one-third of the total deaths. From this disease the mortality among the Native soldiers was nearly four times as great as that among the European troops. On the other hand, the death-rate from enteric fever among the Native troops was only 0·10 per 1,000, as compared with 4·29 among the European troops.

### General Population.

The year 1902 was comparatively dry, the rainfall in India as a whole having been slightly below the annual mean. The deaths from the chief causes, and from all diseases, were as follows:—Cholera 224,135, ratio per 1,000 was 1·01; small-pox 115,603, ratio 0·52; fevers 4,291,952, ratio 19·17; dysentery and diarrhœa 266,869, ratio 1·06; plague 445,293, ratio 2·0; total deaths from all causes 7,112,336, ratio per 1,000 was 31·49.

### Vaccination.

During 1902–1903 there were 8,431,564 persons vaccinated in the several provinces of India, being an increase of

395,120 on the number of the previous year. The increase was spread over all the provinces except the Punjab.

Civil Hospitals and Dispensaries.

There were no fewer than 2,459 hospitals and dispensaries under official control open in India during 1902, and 372,780 in-patients, and 24,039,114 out-patients were treated at the institutions during the year.

Sanitation.

In Calcutta the work of improving clusters of native huts was carried on mainly on the same lines as in previous years. Two hundred and twenty-eight huts were demolished during the year, thereby removing some of the most insanitary plague spots in the city. Progress was made in the extension of the filtered and unfiltered water supply, and the wastage of sullage, though still considerable, has been greatly reduced. The municipalities of the interior of Bengal spent nearly one-half of their income on sanitary works during the financial year 1901-1902. In the Punjab, important works in connection with the water supply and sanitation of several large towns were under construction in 1902. In the Central Provinces, the most important sanitary works of the year were the increase of the storage capacity of the Ambajhari reservoir, which supplies Nagpur with water, and the completion of the Bhandara waterworks. The municipalities of Lower Burma spent 49 per cent. of their income, or 14 per cent. more than in the previous year, on sanitary works. The expenditure on water supply was more than doubled. In Madras city a sum of £22,500 was expended on sanitation during the year. The municipalities of Berar spent 40 per cent. of their income on sanitation, as compared with 36 per cent. in 1901.

